# Long-term incidence of infective endocarditis according to the type of cardiac implantable electronic device: a nationwide comparison with moderate- and high-risk patient groups

**DOI:** 10.1093/europace/euag232

**Published:** 2026-08-28

**Authors:** Amna Alhakak, Lauge Østergaard, Mohammed Bakir Ahmad Lafta, Michael Vinther, Berit Thornvig Philbert, Henning Bundgaard, Lars Køber, Emil Loldrup Fosbøl

**Affiliations:** Department of Cardiology, Copenhagen University Hospital – Rigshospitalet, Blegdamsvej 9, København Ø, Copenhagen 2100, Denmark; Department of Cardiology, Copenhagen University Hospital – Rigshospitalet, Blegdamsvej 9, København Ø, Copenhagen 2100, Denmark; Department of Cardiology, Copenhagen University Hospital – Rigshospitalet, Blegdamsvej 9, København Ø, Copenhagen 2100, Denmark; Department of Cardiology, Copenhagen University Hospital – Rigshospitalet, Blegdamsvej 9, København Ø, Copenhagen 2100, Denmark; Department of Cardiology, Copenhagen University Hospital – Rigshospitalet, Blegdamsvej 9, København Ø, Copenhagen 2100, Denmark; Department of Cardiology, Copenhagen University Hospital – Rigshospitalet, Blegdamsvej 9, København Ø, Copenhagen 2100, Denmark; Department of Clinical Medicine, University of Copenhagen, Copenhagen, Denmark; Department of Cardiology, Copenhagen University Hospital – Rigshospitalet, Blegdamsvej 9, København Ø, Copenhagen 2100, Denmark; Department of Clinical Medicine, University of Copenhagen, Copenhagen, Denmark; Department of Cardiology, Copenhagen University Hospital – Rigshospitalet, Blegdamsvej 9, København Ø, Copenhagen 2100, Denmark; Department of Clinical Medicine, University of Copenhagen, Copenhagen, Denmark

**Keywords:** Pacemaker, Implantable cardioverter-defibrillator, Cardiac resynchronization therapy, Infective endocarditis, Epidemiology

## Abstract

**Aims:**

According to the European Society of Cardiology (ESC) guidelines, patients with a cardiac implantable electronic device (CIED) are considered at moderate risk of infective endocarditis (IE), irrespective of CIED type. However, comparative data across CIED types and IE risk groups are limited. We examined IE rates according to CIED type compared with moderate- and high-risk groups.

**Methods and results:**

Danish nationwide registries were used to identify patients with first-time CIED implantation and reference populations at moderate and high risk of IE (2000–2022), based on the ESC guidelines. The high-risk group included patients with a left-sided prosthetic valve. Patients with prior IE or left-sided prosthetic valve were excluded from the CIED group. The primary outcome was incident IE within 10 years. The study included 84 171 CIED recipients, 127 966 moderate-risk patients, and 23 505 high-risk patients. Pacemakers were the most common CIED type (74.9%), followed by implantable cardioverter-defibrillator (16.6%), cardiac resynchronization therapy device with defibrillator (CRT-D) (4.6%), and cardiac resynchronization therapy device without defibrillator (3.9%). Pacemaker recipients were the oldest (median 78.2 years) and most frail. The 10-year cumulative incidence of IE was lowest in pacemaker recipients (1.0% [95% confidence interval (CI): 0.9–1.1]) and highest in CRT-D recipients (2.2% [95% CI: 1.7–2.9]). In adjusted analyses, all CIED types were associated with higher IE rates than the moderate-risk group but lower than the high-risk group.

**Conclusion:**

In this nationwide cohort, IE risk differed across CIED types, highest in CRT-D and lowest in pacemaker recipients. All CIED types were associated with higher risk than moderate-risk patients but lower risk than high-risk patients, supporting the ESC classification. Cardiac resynchronization therapy device with defibrillator recipients may warrant further evaluation of preventive strategies.

## Graphical Abstract

**Graphical Abstract:**
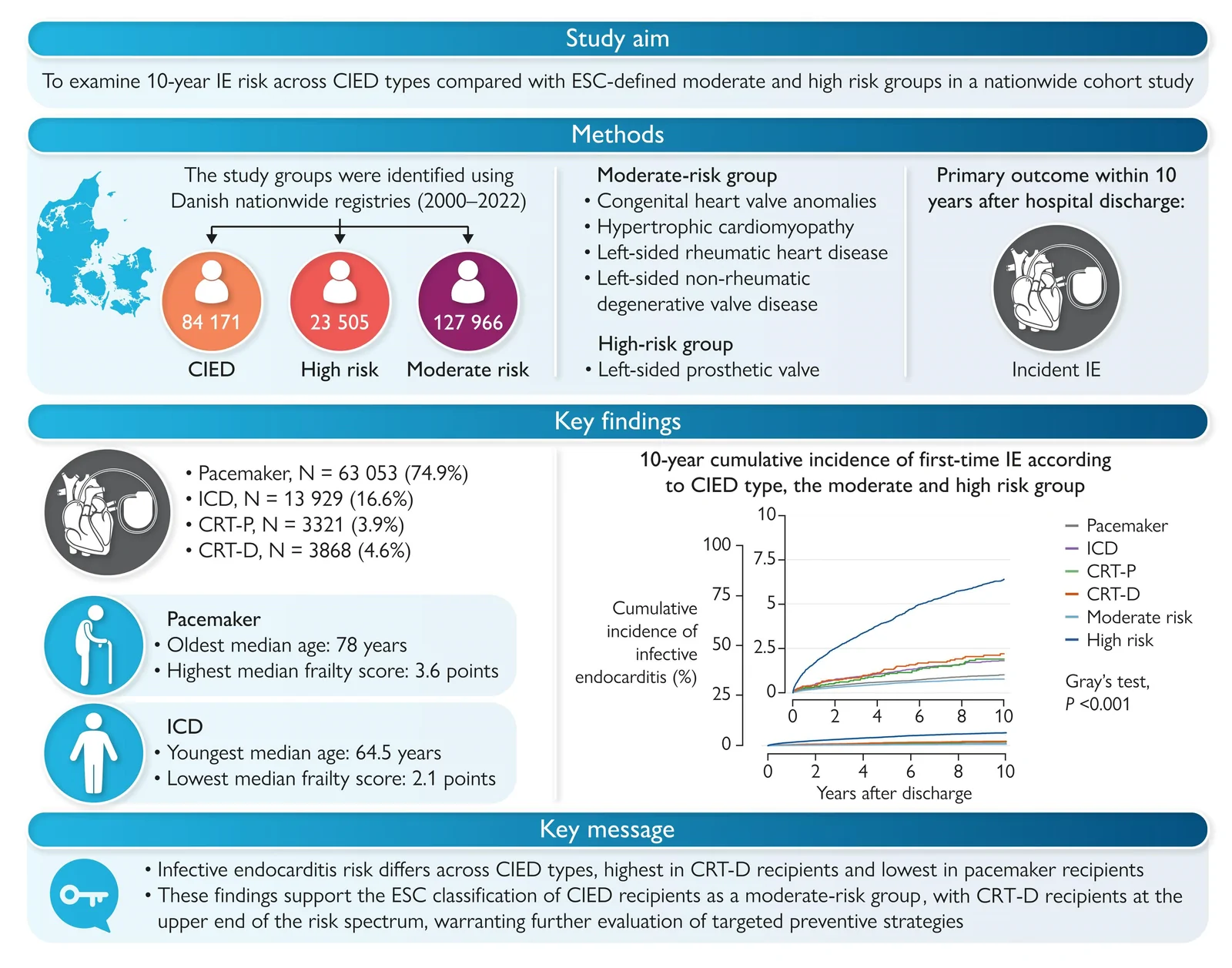
Study design and key findings in CIED recipients vs. ESC-defined reference populations at moderate and high risk of infective endocarditis. Abbreviations: CIED, cardiac implantable electronic device; IE, infective endocarditis. Created with BioRender.com.

What’s new?

- In this nationwide cohort study, the 10-year cumulative incidence of infective endocarditis (IE) differed across cardiac implantable electronic device (CIED) types, ranging from 1.0% in pacemaker recipients to 2.2% in recipients of cardiac resynchronization therapy with defibrillator (CRT-D).
- All CIED types were associated with higher adjusted 10-year IE rates than the European Society of Cardiology (ESC)-defined moderate-risk population, but lower rates than the high-risk population.
- These findings provide nationwide evidence supporting the current ESC classification of CIED recipients as a moderate-risk group for IE.
- Among CIED recipients, CRT-D was associated with the highest estimated long-term risk of IE and may warrant further evaluation of preventive strategies.

## Introduction

According to the 2023 European Society of Cardiology (ESC) guidelines on infective endocarditis (IE), patients with a cardiac implantable electronic device (CIED) are classified as being at moderate risk of IE in relation to preventive strategies.^1^ Antibiotic prophylaxis is recommended before dental or other invasive procedures only in patients at high risk of IE, but not in those at moderate risk, such as patients with a CIED, as the absolute risk is considered very low.^1^

However, information is limited in terms of whether specific CIED types are associated with moderate or high risk of IE, according to pacemaker (PM), implantable cardioverter-defibrillator (ICD), cardiac resynchronization therapy device with defibrillator (CRT-D), or without (CRT-P). A more precise understanding of device-specific IE risk is important to determine whether certain CIED types warrant reassessment within the existing moderate-risk category and to refine preventive strategies, including considerations relevant to antibiotic prophylaxis.

Previous studies have suggested an increasing risk of IE with increasing device complexity (i.e. the number and type of leads), with the highest risk observed in recipients of CRT-D systems, followed by CRT-P, ICD, and PM^2–7^. However, this association remains incompletely characterized across different risk groups, and findings are not entirely consistent.^8,9^ In this context, a large study including more than 1 million patients with a CIED reported the highest infection risk among ICD recipients.^9^ Overall, studies have been limited by the absence of appropriate comparator groups, heterogeneous populations, and variable follow-up.

In this nationwide study, we aimed to examine the 10-year rate of first-time IE in patients after first-time implantation of a CIED, stratified by device type, compared with ESC-defined moderate- and high-risk patient groups in a real-world setting. This approach enables a comprehensive assessment of IE risk after CIED implantation within the broader clinical risk spectrum, defined by the current ESC guidelines.

## Methods

### Data sources

This nationwide cohort study was based on Danish administrative registries, combined at the individual level using the unique personal identification number assigned to all Danish citizens. The Danish National Patient Registry provides data on every hospital contact according to the International Classification of Diseases, 8th Revision (ICD-8) until 1994, and 10th Revision (ICD-10) thereafter, and the Nordic Medico-Statistical Committee Classification of Surgical Procedures (NCSP).^10^ The Danish National Prescription Registry provides information on every dispensed medication from Danish pharmacies based on Anatomical Therapeutic Chemical codes.^11^ The Danish Civil Registration system provides information on sex, birth date, and vital status, such as dates of death and emigration.^12^

### Study exposure

The primary exposure of interest was first-time CIED implantation, categorized as PM, ICD, CRT-P, or CRT-D (see Supplementary material online, *Table S1*). These patients were compared with two non-CIED reference groups at moderate and high risk of IE. The moderate-risk group was defined, based on the ESC guidelines,^1^ as patients with congenital heart valve anomalies, hypertrophic cardiomyopathy (HCM), left-sided rheumatic heart disease, or left-sided non-rheumatic degenerative valve disease (see Supplementary material online, *Table S2*). The high-risk group included patients who had undergone left-sided prosthetic heart valve implantation, including both surgical and transcatheter procedures (e.g. transcatheter aortic valve implantation [TAVI]) (see Supplementary material online, *Table S1*).

To ensure mutually exclusive groups, patients were assigned to a single study group using a predefined hierarchical algorithm. Overlap between CIED exposure and left-sided prosthetic valve surgery was resolved based on the earliest qualifying event, with valve surgery prioritized if both occurred on the same date. Across all eligible groups, patients were subsequently assigned to a final study group according to the predefined hierarchy based on the most recent qualifying event.

### Study population

The study population comprised all patients aged ≥18 years who fulfilled the criteria for the respective study groups and survived to hospital discharge between 1 January 2000 and 31 December 2022. The index date was defined as the date of hospital discharge.

Group assignment was performed prior to applying exclusion criteria.

The CIED group included patients with first-time implanted transvenous CIEDs, excluding those with leadless PMs or subcutaneous ICDs. Patients with left-sided prosthetic valve surgery on or before the index date were excluded from the CIED and moderate-risk groups, as these patients were included in the high-risk group. Patients with CIED implantation on or before the index date were excluded from the moderate- and high-risk groups. Patients with IE on or before the index date were excluded from all groups. In a supplementary analysis, patients with subcutaneous ICDs and leadless PMs were included.

The positive predictive values (PPVs) of the procedure codes for CIEDs and left-sided heart valve surgery were 100%,^13^ while the PPVs for the ICD-10 codes of HCM, congenital heart valve anomalies, and non-rheumatic degenerative valve diseases were 90%,^13^ 98%,^10^ and 96%,^13^ respectively. No validation data for rheumatic heart diseases are available.

### Study outcomes and follow-up

The primary outcome was incident IE, defined as an IE hospital admission of minimum 14 days, or death within 14 days of admission (see Supplementary material online, *Table S2*). The PPV of this IE definition was 90%.^14,15^ The secondary outcome was all-cause mortality.

For the primary outcome, patients were followed from the day after index date until the first occurrence of IE, death, emigration, subsequent left-sided prosthetic heart valve surgery, subsequent CIED procedure (in the CIED group only), or end of study (31 December 2023), with a maximum follow-up of 10 years and a minimum of 1 year. Subsequent left-sided prosthetic heart valve surgery was treated as a censoring event in all study groups, as it was considered to represent a major change in IE risk status, whereas subsequent CIED procedures were only treated as censoring events in the CIED group. To avoid misclassifying the index procedure as a new event, left-sided prosthetic valve surgery in the high-risk group and CIED procedures in the CIED group were only considered censoring events if they occurred >14 days after the index date. Cardiac implantable electronic device type and risk groups were defined at the index date and were not reclassified during follow-up.

For the secondary outcome, patients were followed from the day after the index date until death, emigration, or end of study (31 December 2023), with a maximum follow-up of 10 years.

### Patient characteristics, comorbidity, and pharmacotherapy

Comorbidities were identified based on primary and secondary diagnoses from hospital contacts any time before or on the index date (see Supplementary material online, *Table S2*). Hypertension was identified according to a combination of minimum two dispensed antihypertensive drugs.^16^ Diabetes was identified according to diagnosis codes or dispensed antidiabetic medication.^17,18^ Dialysis was identified based on diagnosis or NCSP code. Concomitant medication was identified based on dispensed drugs within 6 months before or on the index date (see Supplementary material online, *Table S3*).

Frailty at baseline was evaluated using the validated Hospital Frailty Risk Score.^19^ The score was based on hospital admissions within the 10 years preceding the IE admission and was categorized into three levels: low (0–4), intermediate (5–15), and high (>15) frailty risk. Frailty was defined as a score of ≥5.

### Statistical analyses

Baseline characteristics were summarized as frequencies with percentages for categorical variables and medians with interquartile ranges (IQRs; 25th–75th percentiles) for continuous variables.

The Aalen–Johansen estimator was used to calculate the cumulative incidence of incident IE, which accounts for the competing risk of death. Gray’s test was used to assess differences between study groups. The Kaplan–Meier estimator was used to calculate the cumulative incidence of all-cause mortality, and the log-rank test was used to assess differences between study groups.

We used cause-specific Cox regression models for IE and multivariable Cox regression models for all-cause mortality to calculate adjusted hazard ratios (HRs) with 95% confidence intervals (CIs), using the moderate-risk group as the reference. Pairwise comparisons between CIED types were performed within the cause-specific Cox regression model.

The models were adjusted for sex, age (as a continuous variable), stroke, chronic obstructive pulmonary disease, diabetes, cancer, liver disease, chronic renal disease, dialysis, and calendar year. Model assumptions were assessed and met, including the proportional hazards assumption evaluated using Schoenfeld residuals, linear relationships for continuous covariates, and absence of significant interactions between the study groups and clinically relevant variables.

### Sensitivity analyses

Sensitivity analyses included stratified analyses by single-chamber PM, dual-chamber PM, single-chamber ICD, dual-chamber ICD, CRT-P, and CRT-D, as well as analyses of the total CIED group compared with the moderate- and high-risk IE groups.

The ESC-defined moderate-risk population was stratified according to the individual subgroups. Ten-year cumulative incidence curves of IE were estimated for each subgroup. Furthermore, a cause-specific Cox regression analysis was performed using the largest moderate-risk subgroup, non-rheumatic degenerative valve disease, as the reference category.

In additional sensitivity analyses, both cumulative incidence curves and cause-specific Cox models were assessed without censoring at subsequent CIED procedures in CIED recipients.

To assess temporal variation, we performed a supplementary analysis restricted to CIED recipients and tested the interaction between CIED type and index year in the cause-specific Cox regression model, with PM recipients as the reference group. As the overall interaction was statistically significant, the primary adjusted analysis was subsequently repeated stratified by calendar period (2000–2012 and 2013–2022), reflecting changes in CIED technology and implantation practice over time, with the moderate-risk group used as the reference group in both periods.

### Ethical approval

This study was approved by the Capital Region of Denmark, in compliance with the General Data Protection Regulation. Ethical approval is not required for registry-based studies in Denmark using pseudo-anonymized data. Microdata with fewer than three observations were not reported, as required by Danish law, although all authors had access to the precise numbers.

## Results

### Study groups

After fulfilment of the predefined selection criteria, the study population comprised 84 171 patients with a CIED, 127 966 patients in the moderate-risk group, and 23 505 patients in the high-risk group with left-sided prosthetic heart valves. The CIED group included 63 053 with a PM, 13 929 with an ICD, 3321 with a CRT-P, and 3868 with a CRT-D (*Figure 1*). In a supplementary analysis, 52 subcutaneous ICD recipients and 294 leadless PM recipients were identified. The moderate-risk group included 4572 with congenital heart valve anomalies, 4705 with HCM, 2115 with left-sided rheumatic heart disease, and 116 574 with left-sided non-rheumatic degenerative valve disease. The high-risk group included 21 812 patients with aortic and 1693 with mitral prosthetic heart valves.

**Figure 1:**
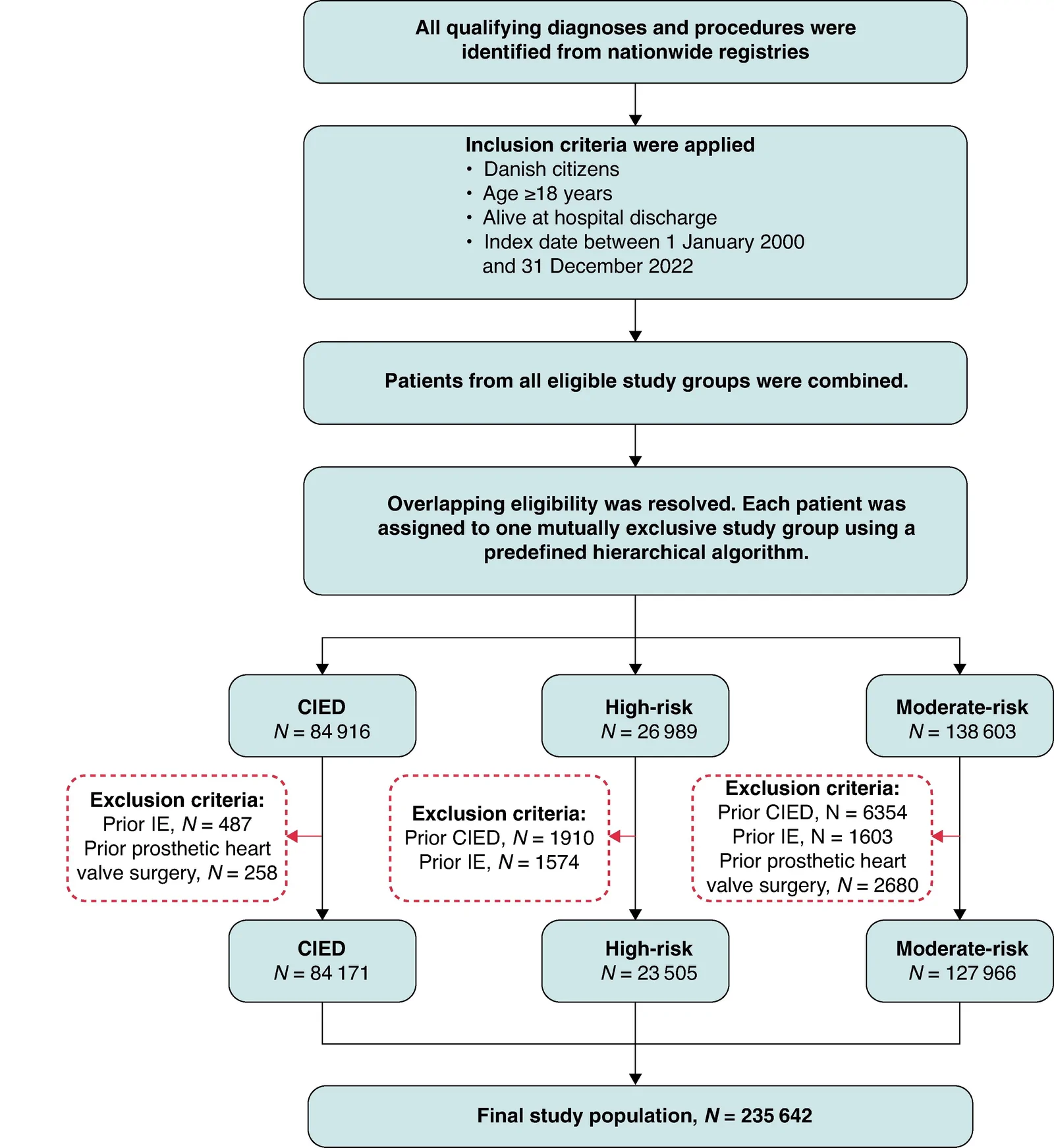
Flowchart of the study population selection and group allocation. Abbreviations: CIED, cardiac implantable electronic device; IE, infective endocarditis.

### Baseline characteristics

Baseline characteristics, stratified by CIED type and the risk groups, are summarized in *Table 1*. Among all CIED types, the prevalence of females was highest in the PM group (42.4%) and lowest in the ICD group (18.8%). Patients with PM had the highest median age (78.2 years [IQR: 70.5–84.5]), whereas patients with ICD had the lowest median age (64.5 years [IQR: 55.3–71.8]). The PM group had the highest median frailty score, whereas the ICD group had the lowest.

**Table 1:** Baseline characteristics according to CIED type, the moderate- and high-risk group of infective endocarditis

Variable	Pacemaker, *N* = 63 053 (%)	ICD, *N* = 13 929 (%)	CRT-P, *N* = 3321 (%)	CRT-D, *N* = 3868 (%)	Moderate risk, *N* = 127 966 (%)	High risk, *N* = 23 505 (%)
Demographics						
Sex (female)	26 720 (42.4)	2617 (18.8)	1015 (30.6)	823 (21.3)	66 555 (52.0)	8704 (37.0)
Age (median [IQR])	78.2 [70.5, 84.5]	64.5 [55.3, 71.8]	73.3 [64.6, 79.8]	67.4 [59.9, 73.4]	75.6 [65.8, 82.9]	73.5 [65.7, 79.4]
Age groups						
<70 years	14 933 (23.7)	9551 (68.6)	1275 (38.4)	2332 (60.3)	43 572 (34.0)	8702 (37.0)
70–80 years	21 156 (33.6)	3813 (27.4)	1240 (37.3)	1402 (36.2)	39 896 (31.2)	9512 (40.5)
>80 years	26 964 (42.8)	565 (4.1)	806 (24.3)	134 (3.5)	44 498 (34.8)	5291 (22.5)
Frailty score (median [IQR])	3.6 [1.4, 7.8]	2.1 [0, 5.0]	2.7 [0.7, 5.6]	2.3 [0.7, 5.1]	2.6 [0.4, 6.2]	2.2 [0.1, 5.0]
Frailty score						
Low	37 687 (59.8)	10 421 (74.8)	2329 (70.1)	2870 (74.2)	87 688 (68.5)	17 520 (74.5)
Intermediate	20 537 (32.6)	3187 (22.9)	864 (26.0)	902 (23.3)	33 131 (25.9)	5405 (23.0)
High	4829 (7.7)	321 (2.3)	128 (3.9)	96 (2.5)	7147 (5.6)	580 (2.5)
Comorbidities						
Ischaemic heart disease	21 024 (33.3)	10 175 (73.0)	1749 (52.7)	2701 (69.8)	35 155 (27.5)	11 876 (50.5)
Heart failure	11 791 (18.7)	9167 (65.8)	3006 (90.5)	3643 (94.2)	26 494 (20.7)	5758 (24.5)
Atrial fibrillation	25 957 (41.2)	3515 (25.2)	1281 (38.6)	1108 (28.6)	29 859 (23.3)	7334 (31.2)
Stroke	8658 (13.7)	1390 (10.0)	346 (10.4)	420 (10.9)	14 681 (11.5)	2413 (10.3)
Peripheral artery disease	3855 (6.1)	956 (6.9)	201 (6.1)	302 (7.8)	7512 (5.9)	1466 (6.2)
Hypertension	34 068 (54.0)	9522 (68.4)	2971 (89.5)	3411 (88.2)	65 832 (51.4)	14 536 (61.8)
Diabetes	11 582 (18.4)	3093 (22.2)	847 (25.5)	1171 (30.3)	18 545 (14.5)	4193 (17.8)
Chronic obstructive pulmonary disease	6712 (10.6)	1315 (9.4)	491 (14.8)	499 (12.9)	14 062 (11.0)	2651 (11.3)
Cancer	10 791 (17.1)	1238 (8.9)	544 (16.4)	417 (10.8)	21 747 (17.0)	3321 (14.1)
Chronic renal disease	3972 (6.3)	733 (5.3)	296 (8.9)	277 (7.2)	7476 (5.8)	1265 (5.4)
Dialysis	806 (1.3)	373 (2.7)	54 (1.6)	96 (2.5)	2011 (1.6)	702 (3.0)
Liver disease	1400 (2.2)	322 (2.3)	89 (2.7)	105 (2.7)	3738 (2.9)	603 (2.6)
Rheumatic disease	4919 (7.8)	733 (5.3)	239 (7.2)	250 (6.5)	10 440 (8.2)	1835 (7.8)
Pharmacotherapy						
Cholesterol-lowering drugs	23 154 (36.7)	8328 (59.8)	1660 (50.0)	2579 (66.7)	46 465 (36.3)	13 049 (55.5)
Beta-blockers	21 962 (34.8)	9024 (64.8)	2563 (77.2)	3055 (79.0)	42 470 (33.2)	10 649 (45.3)
Calcium channel blockers	18 225 (28.9)	2317 (16.6)	417 (12.6)	505 (13.1)	36 223 (28.3)	7015 (29.8)
Renin–angiotensin system inhibitors	29 050 (46.1)	9218 (66.2)	2839 (85.5)	3348 (86.6)	56 381 (44.1)	11 228 (47.8)
Acetylsalicylic acid	18 690 (29.6)	6608 (47.4)	1295 (39.0)	2037 (52.7)	33 763 (26.4)	10 405 (44.3)
Adenosine diphosphate receptor inhibitors	5608 (8.9)	3446 (24.7)	323 (9.7)	791 (20.4)	11 195 (8.7)	3217 (13.7)
Anticoagulants	17 363 (27.5)	3069 (22.0)	1212 (36.5)	1036 (26.8)	22 682 (17.7)	7847 (33.4)

Abbreviations: CIED, cardiac implantable electronic device; CRT-D, cardiac resynchronization therapy device with defibrillator; CRT-P, cardiac resynchronization therapy device without defibrillator; ICD, implantable cardioverter-defibrillator; IQR, interquartile range.

### Incident infective endocarditis

Median follow-up duration ranged from 4.0 to 5.8 years across the study groups. The number of IE events was 502 (0.8%) in PM recipients, 191 (1.4%) in ICD recipients, 45 (1.4%) in CRT-P recipients, 59 (1.5%) in CRT-D recipients, 766 (0.6%) in the moderate-risk group, and 1250 (5.3%) in the high-risk group. No IE events occurred among leadless PM recipients, and fewer than three IE events occurred among subcutaneous ICD recipients. The cumulative incidences of incident IE within 10 years were 1.0% (95% CI: 0.9–1.1) in patients with PM, 1.8% (95% CI: 1.6–2.1) with ICD, 1.9% (95% CI: 1.4–2.5) with CRT-P, and 2.2% (95% CI: 1.7–2.9) with CRT-D, compared with 0.78% (95% CI: 0.72–0.84) in the moderate-risk group and 6.4% (95% CI: 6.1–6.8) in the high-risk group (*Figure 2*).

**Figure 2:**
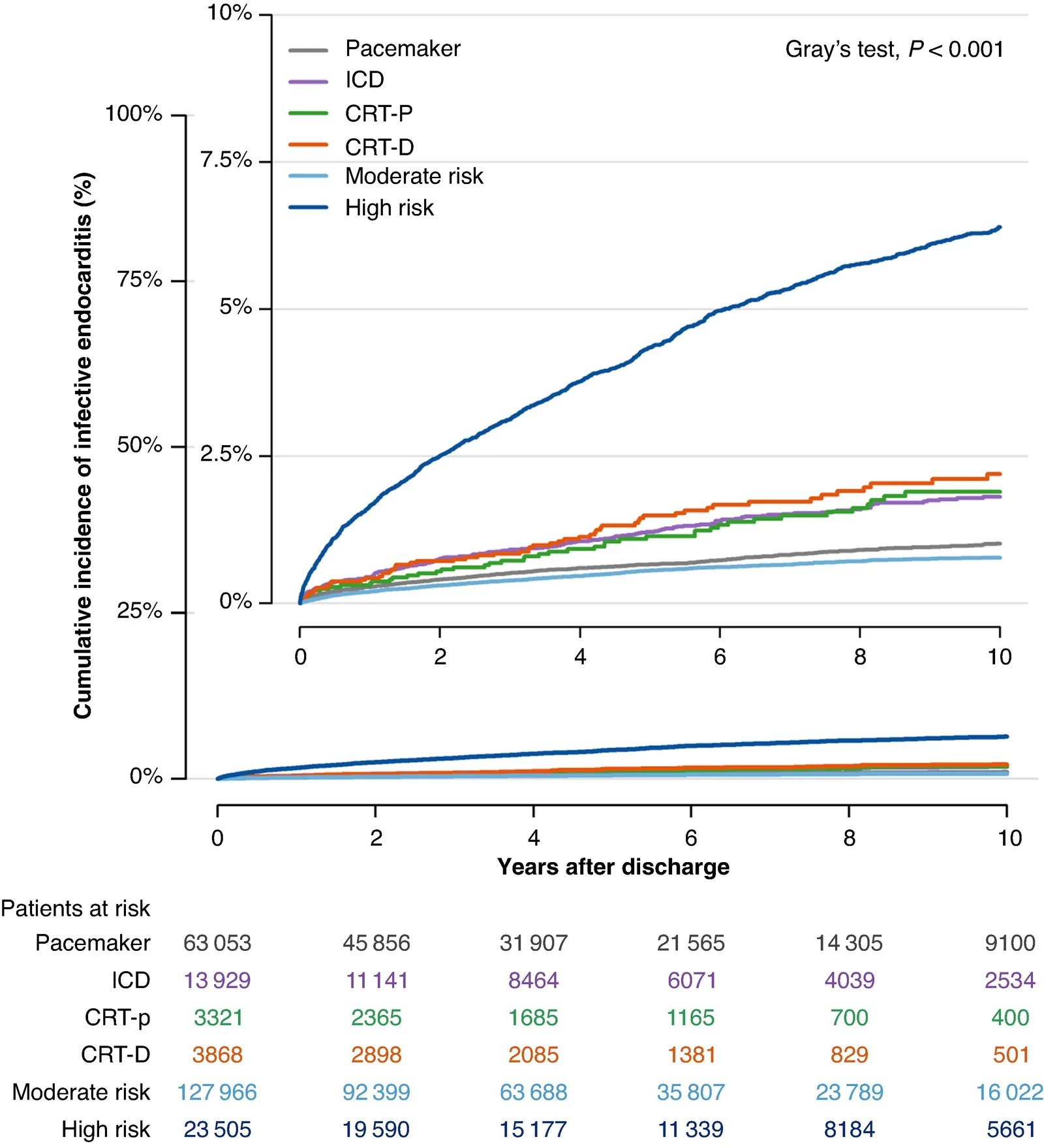
Ten-year cumulative incidence of first-time infective endocarditis (IE) according to CIED type, the moderate- and high-risk group of IE. Abbreviations: CIED, cardiac implantable electronic device; CRT-D, cardiac resynchronization therapy device with defibrillator; CRT-P, cardiac resynchronization therapy device without defibrillator; ICD, implantable cardioverter-defibrillator; IE, infective endocarditis.

In cause-specific Cox regression (*Figure 3*), compared with the moderate-risk group, CRT-D was associated with the highest 10-year rate of IE (adjusted HR: 2.03 [95% CI: 1.56–2.66]), followed by CRT-P and ICD. Patients with CRT-D, CRT-P, and ICD had similar adjusted 10-year IE rates, with no statistically significant differences in pairwise comparisons (all *P* > 0.05). The PM group was associated with a significantly lower IE rate than the other CIED types but a higher rate than the moderate-risk group (adjusted HR: 1.16 [95% CI: 1.03–1.30]). All CIED types had significantly lower IE rates than the high-risk group.

**Figure 3:**
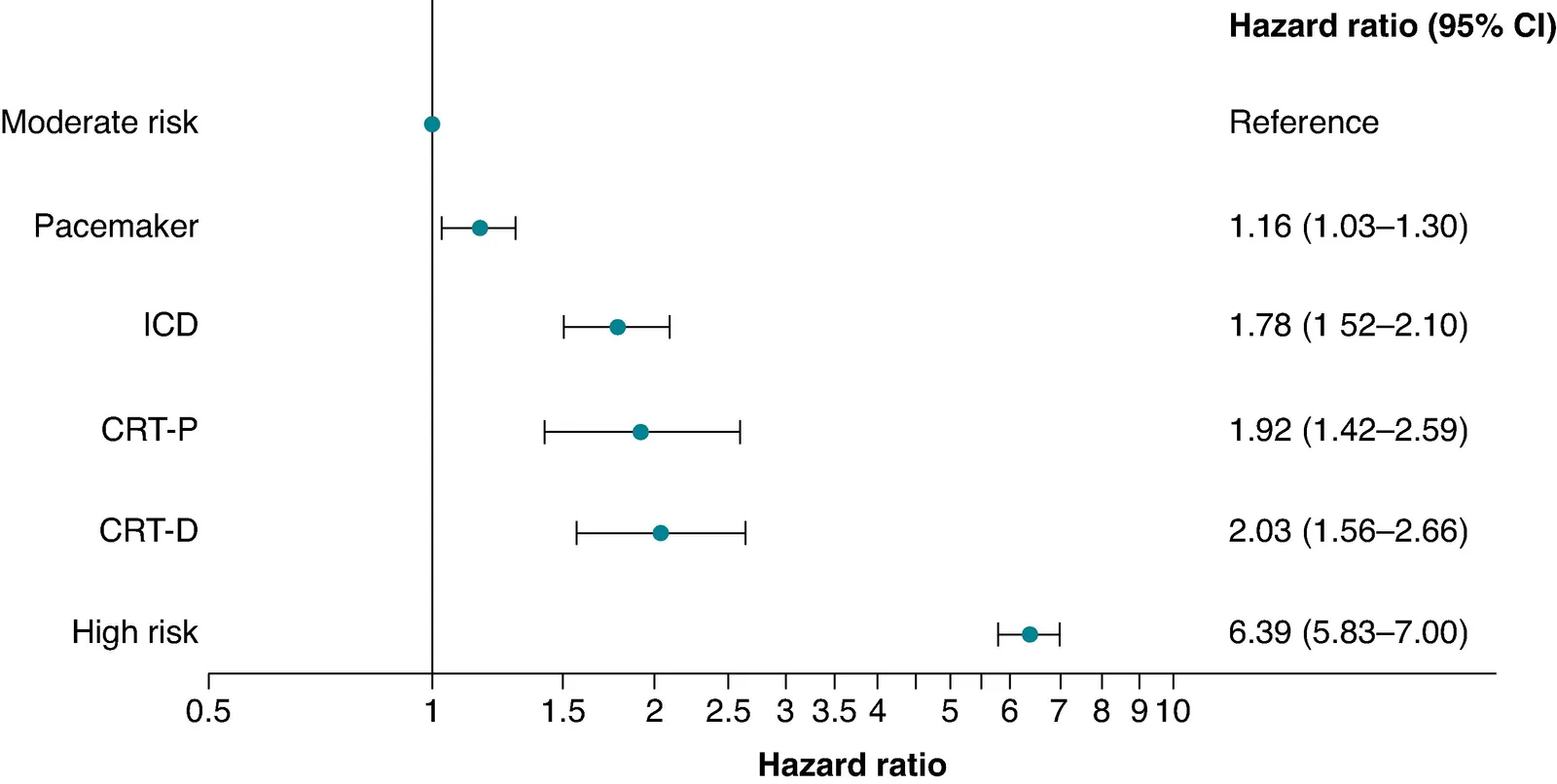
Adjusted 10-year hazard ratios of first-time infective endocarditis (IE) according to CIED type and the high-risk group at baseline compared with the moderate-risk group of IE. The cause-specific Cox regression model was adjusted for sex, age (as a continuous variable), stroke, chronic obstructive pulmonary disease, diabetes, cancer, liver disease, chronic renal disease, dialysis, and calendar year. The error bars represent 95% confidence intervals. Abbreviations: CI, confidence interval; CIED, cardiac implantable electronic device; CRT-D, cardiac resynchronization therapy device with defibrillator; CRT-P, cardiac resynchronization therapy device without defibrillator; ICD, implantable cardioverter-defibrillator

### All-cause mortality

The cumulative incidences of incident all-cause mortality within 10 years were 63.8% (95% CI: 63.3–64.2) in patients with PM, 41.4% (95% CI: 40.4–42.4) with ICD, 60.2% (95% CI: 58.1–62.1) with CRT-P, and 53.7% (95% CI: 51.7–55.7) with CRT-D, compared with 61.5% (95% CI: 61.2–61.9) in the moderate-risk group and 50.0% (95% CI: 49.2–50.8) in the high-risk group (see Supplementary material online, *Figure S1*).

### Sensitivity analyses

In sensitivity analyses, patients with single-chamber PM were the oldest CIED subgroup (median age: 81.2 years) (see Supplementary material online, *Table S4*). The 10-year cumulative incidences of incident IE increased across CIED types, ranging from 0.82% in single-chamber PM to 2.2% in CRT-D (see Supplementary material online, *Figure S2*). In cause-specific Cox models, compared with the moderate-risk group, CRT-D was associated with the highest 10-year IE rate, whereas single-chamber PM was associated with no significant difference in IE rates (see Supplementary material online, *Figure S3*).

In an additional sensitivity analysis, baseline characteristics according to the total CIED group and the risk groups are summarized in Supplementary material online, *Table S5*. The total CIED group had a 10-year cumulative incidence of 1.2% and was associated with a significantly higher 10-year IE rate compared with the moderate-risk group (adjusted HR: 1.34) (see Supplementary material online, *Figures S4*–*S5*).

The 10-year cumulative incidence of IE according to the individual moderate-risk subgroups ranged from 0.57 to 0.87% (see Supplementary material online, *Figure S6*). In a supplementary multivariable analysis using non-rheumatic degenerative valve disease as the reference category, adjusted HRs for the remaining moderate-risk subgroups, the individual CIED types, and the high-risk group remained consistent with the primary findings (see Supplementary material online, *Figure S7*).

During follow-up, 7.0% PM recipients, 16.9% ICD recipients, 14.4% CRT-P recipients, and 17.5% CRT-D recipients were censored due to a subsequent CIED procedure. In the sensitivity analyses without censoring at repeat CIED procedures, the number of IE events remained unchanged, and the overall findings were consistent with the primary analyses (see Supplementary material online, *Figures S8* and *S9*).

The interaction between CIED type and year of implantation was statistically significant (*P* for overall interaction = 0.001). Among the individual interaction terms, temporal variation was observed for CRT-P (*P* < 0.001), whereas no statistically significant interaction was observed for the remaining CIED types. In sensitivity analyses stratified by calendar period, the overall pattern of adjusted HRs for the individual CIED types was broadly consistent with the primary analysis in both periods (2000–2012 and 2013–2022) (see Supplementary material online, *Figures S10* and *S11*).

## Discussion

In this Danish nationwide cohort of patients with CIEDs, compared with ESC-defined reference populations at moderate and high risk of IE, we identified three main findings. First, the 10-year cumulative incidence of IE varied across CIED types, with the highest incidence among patients with CRT-D and the lowest among patients with PMs (*Graphical Abstract*). Second, the CRT-D, CRT-P, and ICD groups were all associated with higher 10-year IE rates compared with the moderate-risk group but showed comparable adjusted IE rates. Third, the PM group was associated with the lowest 10-year cumulative incidence of IE among the CIED types but had the highest 10-year cumulative incidence of mortality.

### Long-term rate of infective endocarditis after cardiac resynchronization therapy device with defibrillator implantation

Patients with CRT-D had the highest 10-year cumulative incidence of IE among all CIED types, consistent with previous studies demonstrating that the risk of IE and CIED infection increases with increasing CIED complexity, with the highest risk in CRT-D systems, followed by CRT-P, ICD, and PM^2–8^. However, the results in the literature have been conflicting.^8,9^ A study including more than 1 million patients with a CIED reported the highest risk of CIED infection in patients with an ICD.^9^ These conflicting findings should be interpreted in the context of differences in comparator groups, follow-up duration, and heterogeneous study populations, which may influence the relative risk estimates and the observed ranking of CIED types.

The underlying patient characteristics may also contribute to the higher IE risk in CRT-D recipients, as they often have advanced heart failure with reduced left ventricular function, increased hospital admissions, and frequent invasive procedures with vascular access,^5,20,21^ leading to a higher susceptibility to IE over time. Although CRT-D recipients had the highest 10-year IE rate of all CIED types, their IE rate remained significantly lower than that of the high-risk group, which may partly reflect that CIED-related IE is predominantly lead related with less extensive valve destruction and a lower burden of left-sided prosthetic material than prosthetic valve endocarditis.^1,22^ However, our registry-based data did not allow determination of whether individual IE episodes were CIED-related. These findings support the current ESC guidelines that classify CIED recipients in the moderate-risk group,^1^ but also suggest that CRT-D recipients are at the upper end of this spectrum, highlighting the need for increased clinical awareness in this subgroup. Further studies are warranted to evaluate whether targeted preventive strategies, including consideration of antibiotic prophylaxis, or earlier diagnostic evaluation may be beneficial, especially in CRT-D recipients.

### Long-term rate of infective endocarditis after implantation of cardiac resynchronization therapy devices with and without defibrillator and implantable cardioverter-defibrillators

Pairwise comparisons demonstrated no statistically significant differences in adjusted 10-year IE rates between CRT-D, CRT-P, and ICD recipients, in line with previous studies.^4,23^ All three CIED types were associated with a significantly higher IE rate than the moderate-risk group but a significantly lower rate than the high-risk group. These findings are consistent with the current ESC guidelines^1^ and previous studies,^24–26^ indicating that implantation of a transvenous CIED system is overall a marker of moderate IE risk, regardless of the specific CIED type.

Our findings suggests that IE risk and preventive strategies are broadly similar for CIED systems providing resynchronization and/or defibrillation therapy. However, important differences in patient characteristics should be considered, as CRT recipients are older and have a higher comorbidity burden, whereas ICD recipients are younger but still frequently have underlying heart failure. Chronic kidney disease has been associated with worse clinical outcomes following CIED infection, underscoring the importance of comorbidity in this patient population.^27^ In addition, differences in device complexity and the need for subsequent interventions may contribute to IE risk across groups. These factors may partly explain the comparable long-term IE risk observed.

### Long-term rate of infective endocarditis and mortality after pacemaker implantation

Patients with PMs had the lowest IE rate among all CIED types, but a 16% higher 10-year IE rate than the moderate-risk group, which was observed alongside a generally less complex PM system with fewer leads and a lower prevalence of advanced heart failure and ischaemic heart disease. Pacemakers were also associated with significantly lower IE rates than CRT-D, CRT-P, and ICD systems. These findings are consistent with previous nationwide studies reporting lower IE risk in PM recipients compared with the risk in patients with ICD or CRT systems.^2–6,23,28^ However, in a large nationwide study by Clémenty *et al.*, no difference was identified in the risk of device infection between single- or dual-chamber PMs and CRT-P.^28^

The increased mortality incidence observed in the PM group may reflect confounding by indication, as this group was the oldest and most frail and had the highest prevalence of stroke and cancer among the CIED types. Due to more advanced age and complex comorbidities with limited life expectancy, patients with PM are less likely to undergo device revision or device extraction in the presence of suspected infection,^3,9^ which may contribute both to underdiagnosis and to more conservative treatment strategies.^1,29^ Additionally, these patients are most likely to die from causes other than IE.^3^ In elderly patients with PM, contemporary decisions regarding invasive diagnostic procedures or device extraction must therefore balance a relatively lower IE risk against a high competing risk of mortality. Overall, these associations underscore that CIED type, age, and comorbidity must be considered together when evaluating long-term prognosis. Further research is needed to determine whether some CIED types are at sufficiently high risk to justify enhanced surveillance or prophylactic strategies.

### Study strengths and limitations

The main strengths of this nationwide study are the completeness of long-term follow-up, with no loss to follow-up, and the unselected data, as the Danish health care system gives tax-funded, equal access to all citizens, irrespective of socioeconomic or insurance status. However, several limitations should be acknowledged. First, as this is an observational study, the findings reflect associations rather than causal relationships. Patients with implanted PMs had the highest mortality incidence, which may be influenced by confounding by indication, as this group was the oldest, the most frail, and had the highest proportion of stroke and cancer compared with other CIED types. Despite this, they had the lowest adjusted IE rate compared with the moderate-risk group. Second, residual confounding cannot be excluded, even though we adjusted for different potential confounders in the cause-specific Cox regression analyses. Differences in heart failure severity, left ventricular function, clinical indication, and other unmeasured patient- and procedure-related factors may have influenced the observed associations. Third, misclassification may have affected our findings, despite previously reported PPVs of 90–100% for all study groups. Fourth, the moderate-risk group represents a heterogeneous population with substantial clinical variability, including differences in disease severity, which may have influenced results. Finally, information on several clinically relevant variables was not available, including antibiotic treatment during hospitalization, microbial aetiology across the entire follow-up period, imaging findings, IE phenotype, involvement of the generator and leads, lead technology, the use of antibacterial envelopes, and IE-related complications. Consequently, distinction between isolated pocked infection, pocked-related IE, lead-related IE, and valvular IE was not possible. Furthermore, we were unable to determine whether individual IE episodes were CIED-related. As these represent distinct clinical entities with different clinical presentation and prognosis, this should be considered when interpreting our findings.^30^

## Conclusions

In this Danish nationwide cohort of patients with CIEDs, the 10-year cumulative incidence of IE differed across CIED types, with the lowest incidence among patients with PMs (1.0%) and the highest among those with CRT-D (2.2%). All CIED types were associated with higher adjusted 10-year IE rates than the moderate-risk group but lower rates than the high-risk group.

These findings support the current ESC guidelines classifying CIED recipients as a moderate-risk group without routine antibiotic prophylaxis. However, CRT-D recipients were at the upper end of the risk spectrum within the CIED population. Further studies are warranted to determine whether enhanced surveillance or targeted preventive strategies, including prophylactic interventions, may be beneficial in CRT-D recipients.

## Supplementary Material

euag232_Supplementary_Data

## Data Availability

Data are not publicly available and can be accessed through a third party. Access to the underlying data is granted through Statistics Denmark, subject to Danish data protection regulations.
