# Supplementary material for: Long-term incidence of infective endocarditis according to the type of cardiac implantable electronic device: a nationwide comparison with moderate- and high-risk patient groups
Source: Europace. 2026 Aug 28;28(10):euag232. doi: 10.1093/europace/euag232 (PMC13659775; doi:10.1093/europace/euag232)
Supplement: Supplementary file 1 [file euag232_supplementary_data.docx]

**Supplementary Appendix:**

**Alhakak et al.**

**Title:** Long-term incidence of infective endocarditis according to the type of cardiac implantable electronic device: a nationwide comparison with moderate- and high-risk patient groups

**Contents**

[Table S1. Surgical procedures, NOMESCO Classification of Surgical Procedures (NCSP)	3](#_Toc239687192)

[Table S2. ICD-8 and - 10 codes	3](#_Toc239687193)

[Table S3. Pharmacotherapy, ATC classification codes	6](#_Toc239687194)

[Table S4. Baseline characteristics according to single- or dual-chamber PM, single- or dual-chamber ICD, CRT-P, CRT-D, the moderate- and high-risk group of infective endocarditis.	7](#_Toc239687195)

[Table S5. Baseline characteristics according to the total CIED-group, the moderate- and high-risk group of infective endocarditis.	11](#_Toc239687196)

[Figure S1. 10-year cumulative incidence of all-cause mortality according to CIED type, the moderate- and high-risk group of infective endocarditis.	14](#_Toc239687197)

[Figure S2. 10-year cumulative incidence of first-time infective endocarditis according to single- or dual-chamber PM, single- or dual-chamber ICD, CRT-P, CRT-D, the moderate- and high-risk group of infective endocarditis.	15](#_Toc239687198)

[Figure S3. Adjusted 10-year hazard ratios of first-time infective endocarditis (IE) according single- or dual-chamber PM, single- or dual-chamber ICD, CRT-P, CRT-D, and the high-risk group compared with the moderate-risk group of IE.	16](#_Toc239687199)

[Figure S4. 10-year cumulative incidence of first-time infective endocarditis (IE) according to the total CIED-group, the moderate- and high-risk group of IE.	17](#_Toc239687200)

[Figure S5. Adjusted 10-year hazard ratios of first-time infective endocarditis (IE) according to the total CIED-group and the high-risk group compared with the moderate-risk group of IE.	18](#_Toc239687201)

[Supplementary Figure S6. 10-year cumulative incidence of first-time infective endocarditis (IE) according to CIED type, the individual moderate risk subgroups, and high-risk group of IE.	19](#_Toc239687202)

[Supplementary Figure S7. Adjusted 10-year hazard ratios of first-time infective endocarditis (IE) according to CIED type, the individual moderate risk subgroups, and the high-risk group at baseline compared with the non-rheumatic degenerative valve disease-group	20](#_Toc239687203)

[Supplementary Figure S8. 10-year cumulative incidence of first-time infective endocarditis (IE) according to CIED type, the moderate- and high-risk group of IE without censoring at subsequent CIED procedures in CIED recipients.	21](#_Toc239687204)

[Supplementary Figure S9. Adjusted 10-year hazard ratios of first-time infective endocarditis (IE) according to CIED type and the high-risk group at baseline compared with the moderate-risk group of IE without censoring at subsequent CIED procedures in CIED recipients.	22](#_Toc239687205)

[Supplementary Figure S10: Adjusted hazard ratios of first-time infective endocarditis according to CIED type, 2000–2012	23](#_Toc239687206)

[Supplementary Figure S11: Adjusted hazard ratios of first-time infective endocarditis according to CIED type, 2013–2022	24](#_Toc239687207)

# **Table S1. Surgical procedures, NOMESCO Classification of Surgical Procedures (NCSP)**

|  | **NCSP** |
| --- | --- |
| **Study population** |  |
| **CIED** | BFCA0, BFCB0 (excluding BFCA08 and BFB04) |
| **Exclusion criteria** |  |
| **Prosthetic aortic valve surgery** | KFMD |
| **Prosthetic mitral valve surgery** | KFKD |

*CABG,* *coronary artery bypass grafting; CIED, cardiac implantable electronic device*

# **Table S2. ICD-8 and - 10 codes**

|  | **ICD-8 codes** | **ICD-10 codes** |
| --- | --- | --- |
| **Study population** |  |  |
| **Hypertrophic cardiomyopathy** | 42599 | I421, I422 |
| **Congenital heart valve anomalies** | 74660-74663 | Q22, Q23 |
| **Rheumatic heart diseases** | 393-398 | I05-I06 |
| **Non-rheumatic degenerative valve diseases** | 394-396 | I34-I35 |
|  |  |  |
| **Outcome** |  |  |
| **Infective endocarditis** | 421 | I33, I38, I398 |
| **Comorbidities** |  |  |
| **Ischaemic heart disease** | 410-414 | I20-I25 |
| **Heart failure** | 425, 428, 4270, 4271 | I42, I50, I110, I130, I132, J819 |
| **Atrial fibrillation** | 42793, 42794 | I48 |
| **Stroke** | 430-434, 436 | I60-164 |
| **Peripheral artery** **disease** | 440, 444 | I70, I74 |
| **Diabetes** | 250 | E10-14  or ATC code A10 |
| **COPD** | 490-492 | J42-J44 |
| **Cancer** | 140-209 (if not 173) | C00-C97 (if not C44) |
| **Liver disease** | 571-573, 155, 070 | B15-B19, K70-K77, C22, I982, Z944, D684C, Q618A |
| **Rheumatic disease** | 7100, 7101, 7104, 7140-7142, 7148, 725 | M05, M06, M32-M34, M353 |
| **Chronic kidney disease** | 403, 404, 581-584, 25002, 40039, 59009, 59320, 75310, 75311, 75319 | E102, E112, E132, E142, I120, N02-N08, N11, N12, N14, N18, N19, N26, N158-N160, N162-N164, N168, M300, M313, M319, M321B, Q612, Q613, Q615, Q619, T858, T859 |
| **Dialysis** |  | Z992  or procedure code BJFD |

*ATC, Anatomical Therapeutic Chemical;
COPD, chronic obstructive pulmonary disease;
ICD-8, 8^th^ revision of the International Classification of Diseases;
ICD-10, 10^th^ revision of the International Classification of Diseases;*

*NCSP, NOMESCO Classification of Surgical Procedures*

# **Table S3. Pharmacotherapy, ATC classification codes**

| **Pharmacotherapy** | **ATC codes** |
| --- | --- |
| **Cholesterol-lowering drugs** | C10 |
| **Beta-blockers** | C07, C09BX |
| **Calcium channel blockers** | C07F, C08, C09BB, C09DB |
| **Renin–angiotensin system inhibitors** | C09 |
| **Thiazides** | C03A, C03EA01, C07B, C07D, C09XA52 |
| **Mineralocorticoid receptor antagonists** | C03DA01, C03DA02, C03DA03, C03DA04 |
| **Loop diuretics** | C03C, C03EB01, C03EB02 |
| **Antiadrenergic drugs** | C02A, C02B, C02C |
| **Vasodilators** | C02DB, C02DD, C02DG |
| **Acetylsalicylic acid** | B01AC06 |
| **Adenosine diphosphate receptor inhibitors** | B01AC04, B01AC22, B01AC24 |
| **Anticoagulants** | B01AA, B01AE, B01AF |
| **Hypertension** | Beta-blockers, calcium channel blockers, renin–angiotensin system inhibitors, thiazides, mineralocorticoid receptor antagonists, loop diuretics, antiadrenergic drugs, and vasodilators. |

*ATC, Anatomical Therapeutic Chemical.*

# **Table S4. Baseline characteristics according to single- or dual-chamber PM, single- or dual-chamber ICD, CRT-P, CRT-D, the moderate- and high-risk group of infective endocarditis.**

| **Variable** | **Single-chamber pacemaker, N=22,536 (%)** | **Dual-chamber pacemaker, N=40,517 (%)** | **Single-chamber ICD, N=11,043 (%)** | **Dual-chamber ICD, N=2,886 (%)** | **CRT-P, N=3,321 (%)** | **CRT-D, N=3,868 (%)** | **Moderate risk, N=127,966 (%)** | **High risk, N=23,505 (%)** |
| --- | --- | --- | --- | --- | --- | --- | --- | --- |
| **Demographics** |  |  |  |  |  |  |  |  |
| Sex (Female) | 9,797 (43.5) | 16,923 (41.8) | 2,009 (18.2) | 608 (21.1) | 1,015 (30.6) | 823 (21.3) | 66,555 (52.0) | 8,704 (37.0) |
| Age (median [IQR]) | 81.2 [73.9, 87.0] | 76.6 [68.9, 82.8] | 64.2 [55.0, 71.6] | 65.7 [56.6, 72.9] | 73.3 [64.6, 79.8] | 67.4 [59.9, 73.4] | 75.6 [65.8, 82.9] | 73.5 [65.7, 79.4] |
| **Age groups** |  |  |  |  |  |  |  |  |
| <70 years | 3,692 (16.4) | 11,241 (27.7) | 7,671 (69.5) | 1,880 (65.1) | 1,275 (38.4) | 2,332 (60.3) | 43,572 (34.0) | 8,702 (37.0) |
| 70-80 years | 6,512 (28.9) | 14,644 (36.1) | 2,947 (26.7) | 866 (30.0) | 1,240 (37.3) | 1,402 (36.2) | 39,896 (31.2) | 9,512 (40.5) |
| >80 years | 12,332 (54.7) | 14,632 (36.1) | 425 (3.8) | 140 (4.9) | 806 (24.3) | 134 (3.5) | 44,498 (34.8) | 5,291 (22.5) |
| Frailty score (median [IQR]) | 4.4 [1.8, 9.1] | 3.4 [1.2, 7.0] | 2.1 [0, 5.0] | 2.1 [0.4, 4.8] | 2.7 [0.7, 5.6] | 2.3 [0.7, 5.1] | 2.6 [0.4, 6.2] | 2.2 [0.1, 5.0] |
| **Frailty score** |  |  |  |  |  |  |  |  |
| Low | 12,147 (53.9) | 25,540 (63.0) | 8,240 (74.6) | 2,181 (75.6) | 2,329 (70.1) | 2,870 (74.2) | 87,688 (68.5) | 17,520 (74.5) |
| Intermediate | 8,017 (35.6) | 12,520 (30.9) | 2,547 (23.1) | 640 (22.2) | 864 (26.0) | 902 (23.3) | 33,131 (25.9) | 5,405 (23.0) |
| High | 2,372 (10.5) | 2,457 (6.1) | 256 (2.3) | 65 (2.3) | 128 (3.9) | 96 (2.5) | 7,147 (5.6) | 580 (2.5) |
| **Comorbidities** |  |  |  |  |  |  |  |  |
| Ischemic heart disease | 8,092 (35.9) | 12,932 (31.9) | 8,199 (74.2) | 1,976 (68.5) | 1,749 (52.7) | 2,701 (69.8) | 35,155 (27.5) | 11,876 (50.5) |
| Heart failure | 5,771 (25.6) | 6,020 (14.9) | 7,353 (66.6) | 1,814 (62.9) | 3,006 (90.5) | 3,643 (94.2) | 26,494 (20.7) | 5,758 (24.5) |
| Atrial fibrillation | 13,561 (60.2) | 12,396 (30.6) | 2,627 (23.8) | 888 (30.8) | 1,281 (38.6) | 1,108 (28.6) | 29,859 (23.3) | 7,334 (31.2) |
| Stroke | 3,736 (16.6) | 4,922 (12.1) | 1,101 (10.0) | 289 (10.0) | 346 (10.4) | 420 (10.9) | 14,681 (11.5) | 2,413 (10.3) |
| Peripheral artery disease | 1,709 (7.6) | 2,146 (5.3) | 745 (6.7) | 211 (7.3) | 201 (6.1) | 302 (7.8) | 7,512 (5.9) | 1,466 (6.2) |
| Hypertension | 13,407 (59.5) | 20,661 (51.0) | 7,644 (69.2) | 1,878 (65.1) | 2,971 (89.5) | 3,411 (88.2) | 65,832 (51.4) | 14,536 (61.8) |
| Diabetes | 4,315 (19.1) | 7,267 (17.9) | 2,518 (22.8) | 575 (19.9) | 847 (25.5) | 1,171 (30.3) | 18,545 (14.5) | 4,193 (17.8) |
| Chronic obstructive pulmonary disease | 2,675 (11.9) | 4,037 (10.0) | 1,054 (9.5) | 261 (9.0) | 491 (14.8) | 499 (12.9) | 14,062 (11.0) | 2,651 (11.3) |
| Cancer | 4,193 (18.6) | 6,598 (16.3) | 984 (8.9) | 254 (8.8) | 544 (16.4) | 417 (10.8) | 21,747 (17.0) | 3,321 (14.1) |
| Chronic renal disease | 1,588 (7.0) | 2,384 (5.9) | 578 (5.2) | 155 (5.4) | 296 (8.9) | 277 (7.2) | 7,476 (5.8) | 1,265 (5.4) |
| Dialysis | 337 (1.5) | 469 (1.2) | 297 (2.7) | 76 (2.6) | 54 (1.6) | 96 (2.5) | 2,011 (1.6) | 702 (3.0) |
| Liver disease | 473 (2.1) | 927 (2.3) | 258 (2.3) | 64 (2.2) | 89 (2.7) | 105 (2.7) | 3,738 (2.9) | 603 (2.6) |
| Rheumatic disease | 1,749 (7.8) | 3,170 (7.8) | 559 (5.1) | 174 (6.0) | 239 (7.2) | 250 (6.5) | 10,440 (8.2) | 1,835 (7.8) |
| **Pharmacotherapy** |  |  |  |  |  |  |  |  |
| Cholesterol-lowering drugs | 7,523 (33.4) | 15,631 (38.6) | 6,726 (60.9) | 1,602 (55.5) | 1,660 (50.0) | 2,579 (66.7) | 46,465 (36.3) | 13,049 (55.5) |
| Beta blockers | 8,726 (38.7) | 13,236 (32.7) | 7,262 (65.8) | 1,762 (61.1) | 2,563 (77.2) | 3,055 (79.0) | 42,470 (33.2) | 10,649 (45.3) |
| Calcium channel blockers | 6,619 (29.4) | 11,606 (28.6) | 1,801 (16.3) | 516 (17.9) | 417 (12.6) | 505 (13.1) | 36,223 (28.3) | 7,015 (29.8) |
| Renin angiotensin system inhibitors | 10,649 (47.3) | 18,401 (45.4) | 7,415 (67.1) | 1,803 (62.5) | 2,839 (85.5) | 3,348 (86.6) | 56,381 (44.1) | 11,228 (47.8) |
| Acetylsalicylic acid | 6,564 (29.1) | 12,126 (29.9) | 5,308 (48.1) | 1,300 (45.0) | 1,295 (39.0) | 2,037 (52.7) | 33,763 (26.4) | 10,405 (44.3) |
| Adenosine diphosphate receptor inhibitors | 1,797 (8.0) | 3,811 (9.4) | 2,912 (26.4) | 534 (18.5) | 323 (9.7) | 791 (20.4) | 11,195 (8.7) | 3,217 (13.7) |
| Anticoagulants | 8,902 (39.5) | 8,461 (20.9) | 2,443 (22.1) | 626 (21.7) | 1,212 (36.5) | 1,036 (26.8) | 22,682 (17.7) | 7,847 (33.4) |

Abbreviations: CRT-P, cardiac resynchronization therapy device without defibrillator; CRT-D, cardiac resynchronization therapy device with defibrillator; ICD, implantable cardioverter-defibrillator; IQR, interquartile range

# **Table S5. Baseline characteristics according to the total CIED-group, the moderate- and high-risk group of infective endocarditis.**

| **Variable** | **CIED group, N=84,171 (%)** | **Moderate risk, N=127,966 (%)** | **High risk, N=23,505 (%)** |
| --- | --- | --- | --- |
| **Demographics** |  |  |  |
| Sex (Female) | 31,175 (37.0) | 66,555 (52.0) | 8,704 (37.0) |
| Age (median [IQR]) | 75.4 [66.5, 82.7] | 75.6 [65.8, 82.9] | 73.5 [65.7, 79.4] |
| **Age groups** |  |  |  |
| <70 years | 28,091 (33.4) | 43,572 (34.0) | 8,702 (37.0) |
| 70-80 years | 27,611 (32.8) | 39,896 (31.2) | 9,512 (40.5) |
| >80 years | 28,469 (33.8) | 44,498 (34.8) | 5,291 (22.5) |
| Frailty score (median [IQR]) | 3.3 [1.1, 7.0] | 2.6 [0.4, 6.2] | 2.2 [0.1, 5.0] |
| **Frailty score** |  |  |  |
| Low | 53,307 (63.3) | 87,688 (68.5) | 17,520 (74.5) |
| Intermediate | 25,490 (30.3) | 33,131 (25.9) | 5,405 (23.0) |
| High | 5,374 (6.4) | 7,147 (5.6) | 580 (2.5) |
| **Comorbidities** |  |  |  |
| Ischemic heart disease | 35,649 (42.4) | 35,155 (27.5) | 11,876 (50.5) |
| Heart failure | 27,607 (32.8) | 26,494 (20.7) | 5,758 (24.5) |
| Atrial fibrillation | 31,861 (37.9) | 29,859 (23.3) | 7,334 (31.2) |
| Stroke | 10,814 (12.8) | 14,681 (11.5) | 2,413 (10.3) |
| Peripheral artery disease | 5,314 (6.3) | 7,512 (5.9) | 1,466 (6.2) |
| Hypertension | 49,972 (59.4) | 65,832 (51.4) | 14,536 (61.8) |
| Diabetes | 16,693 (19.8) | 18,545 (14.5) | 4,193 (17.8) |
| Chronic obstructive pulmonary disease | 9,017 (10.7) | 14,062 (11.0) | 2,651 (11.3) |
| Cancer | 12,990 (15.4) | 21,747 (17.0) | 3,321 (14.1) |
| Chronic renal disease | 5,278 (6.3) | 7,476 (5.8) | 1,265 (5.4) |
| Dialysis | 1,329 (1.6) | 2,011 (1.6) | 702 (3.0) |
| Liver disease | 1,916 (2.3) | 3,738 (2.9) | 603 (2.6) |
| Rheumatic disease | 6,141 (7.3) | 10,440 (8.2) | 1,835 (7.8) |
| **Pharmacotherapy** |  |  |  |
| Cholesterol-lowering drugs | 35,721 (42.4) | 46,465 (36.3) | 13,049 (55.5) |
| Beta blockers | 36,604 (43.5) | 42,470 (33.2) | 10,649 (45.3) |
| Calcium channel blockers | 21,464 (25.5) | 36,223 (28.3) | 7,015 (29.8) |
| Renin angiotensin system inhibitors | 44,455 (52.8) | 56,381 (44.1) | 11,228 (47.8) |
| Acetylsalicylic acid | 28,630 (34.0) | 33,763 (26.4) | 10,405 (44.3) |
| Adenosine diphosphate receptor inhibitors | 10,168 (12.1) | 11,195 (8.7) | 3,217 (13.7) |
| Anticoagulants | 22,680 (26.9) | 22,682 (17.7) | 7,847 (33.4) |

Abbreviations: CIED, cardiac implantable electronic device; IQR, interquartile range

# **Figure S1. 10-year cumulative incidence of all-cause mortality according to CIED type, the moderate- and high-risk group of infective endocarditis.**

Abbreviations: CRT-P, cardiac resynchronization therapy device without defibrillator; CRT-D, cardiac resynchronization therapy device with defibrillator; ICD, implantable cardioverter-defibrillator

# **Figure S2. 10-year cumulative incidence of first-time infective endocarditis according to single- or dual-chamber PM, single- or dual-chamber ICD, CRT-P, CRT-D, the moderate- and high-risk group of infective endocarditis.**

Abbreviations: CRT-P, cardiac resynchronization therapy device without defibrillator; CRT-D, cardiac resynchronization therapy device with defibrillator; ICD, implantable cardioverter-defibrillator; IE, infective endocarditis

# **Figure S3. Adjusted 10-year hazard ratios of first-time infective endocarditis (IE) according single- or dual-chamber PM, single- or dual-chamber ICD, CRT-P, CRT-D, and the high-risk group compared with the moderate-risk group of IE.**

The cause-specific Cox regression model was adjusted for sex, age (as a continuous variable), stroke, chronic obstructive pulmonary disease, diabetes, cancer, liver disease, chronic renal disease, dialysis, and calendar year. The error bars represent 95% confidence intervals.

Abbreviations: CI, confidence interval; CRT-P, cardiac resynchronization therapy device without defibrillator; CRT-D, cardiac resynchronization therapy device with defibrillator; ICD, implantable cardioverter-defibrillator

# **Figure S4. 10-year cumulative incidence of first-time infective endocarditis (IE) according to the total CIED-group, the moderate- and high-risk group of IE.**

Abbreviations: CIED, cardiac implantable electronic device; IE, infective endocarditis

# **Figure S5. Adjusted 10-year hazard ratios of first-time infective endocarditis (IE) according to the total CIED-group and the high-risk group compared with the moderate-risk group of IE.**

The cause-specific Cox regression model was adjusted for sex, age (as a continuous variable), stroke, chronic obstructive pulmonary disease, diabetes, cancer, liver disease, chronic renal disease, dialysis, and calendar year. The error bars represent 95% confidence intervals.

Abbreviations: CI, confidence interval; CIED, cardiac implantable electronic device

# **Supplementary Figure S6. 10-year cumulative incidence of first-time infective endocarditis (IE) according to CIED type, the individual moderate risk subgroups, and high-risk group of IE.**

Abbreviations: CRT-P, cardiac resynchronization therapy device without defibrillator; CRT-D, cardiac resynchronization therapy device with defibrillator; ICD, implantable cardioverter-defibrillator; IE, infective endocarditis

# **Supplementary Figure S7. Adjusted 10-year hazard ratios of first-time infective endocarditis (IE) according to CIED type, the individual moderate risk subgroups, and the high-risk group at baseline compared with the non-rheumatic degenerative valve disease-group**

The cause-specific Cox regression model was adjusted for sex, age (as a continuous variable), stroke, chronic obstructive pulmonary disease, diabetes, cancer, liver disease, chronic renal disease, dialysis, and calendar year. The error bars represent 95% confidence intervals. CI, confidence interval

Abbreviations: CI, confidence interval; CRT-P, cardiac resynchronization therapy device without defibrillator; CRT-D, cardiac resynchronization therapy device with defibrillator; ICD, implantable cardioverter-defibrillator

# **Supplementary Figure S8. 10-year cumulative incidence of first-time infective endocarditis (IE) according to CIED type, the moderate- and high-risk group of IE without censoring at subsequent CIED procedures in CIED recipients.**

Abbreviations: CRT-P, cardiac resynchronization therapy device without defibrillator; CRT-D, cardiac resynchronization therapy device with defibrillator; ICD, implantable cardioverter-defibrillator; IE, infective endocarditis

# **Supplementary Figure S9. Adjusted 10-year hazard ratios of first-time infective endocarditis (IE) according to CIED type and the high-risk group at baseline compared with the moderate-risk group of IE without censoring at subsequent CIED procedures in CIED recipients.**

The cause-specific Cox regression model was adjusted for sex, age (as a continuous variable), stroke, chronic obstructive pulmonary disease, diabetes, cancer, liver disease, chronic renal disease, dialysis, and calendar year. The error bars represent 95% confidence intervals. CI, confidence interval

Abbreviations: CI, confidence interval; CRT-P, cardiac resynchronization therapy device without defibrillator; CRT-D, cardiac resynchronization therapy device with defibrillator; ICD, implantable cardioverter-defibrillator

# **Supplementary Figure S10: Adjusted hazard ratios of first-time infective endocarditis according to CIED type, 2000–2012**

The cause-specific Cox regression model was adjusted for sex, age (as a continuous variable), stroke, chronic obstructive pulmonary disease, diabetes, cancer, liver disease, chronic renal disease, dialysis, and calendar year. The error bars represent 95% confidence intervals. CI, confidence interval

Abbreviations: CI, confidence interval; CRT-P, cardiac resynchronization therapy device without defibrillator; CRT-D, cardiac resynchronization therapy device with defibrillator; ICD, implantable cardioverter-defibrillator

# **Supplementary Figure S11: Adjusted hazard ratios of first-time infective endocarditis according to CIED type, 2013–2022**

The cause-specific Cox regression model was adjusted for sex, age (as a continuous variable), stroke, chronic obstructive pulmonary disease, diabetes, cancer, liver disease, chronic renal disease, dialysis, and calendar year. The error bars represent 95% confidence intervals. CI, confidence interval

Abbreviations: CI, confidence interval; CRT-P, cardiac resynchronization therapy device without defibrillator; CRT-D, cardiac resynchronization therapy device with defibrillator; ICD, implantable cardioverter-defibrillator
